# Multi-Omics Analysis Reveals Biaxial Regulatory Mechanisms of Cardiac Adaptation by Specialized Racing Training in Yili Horses

**DOI:** 10.3390/biology14111609

**Published:** 2025-11-17

**Authors:** Tongliang Wang, Mengying Li, Wanlu Ren, Jun Meng, Xinkui Yao, Hongzhong Chu, Runchen Yao, Manjun Zhai, Yaqi Zeng

**Affiliations:** 1 College of Animal Science, Xinjiang Agricultural University, Urumqi 830052, China; wtl13639911402@163.com (T.W.); 17613305908@163.com (M.L.); renwanlu@xjau.edu.cn (W.R.); junm86@xjau.edu.cn (J.M.); yaoxinkui@xjau.edu.cn (X.Y.); zhaimanjun@yeah.net (M.Z.); 2 Xinjiang Key Laboratory of Horse Breeding and Exercise Physiology, Urumqi 830052, China; 3 Horse Industry Research Institute, Xinjiang Agricultural University, Urumqi 830052, China; 4 Xinjiang Yili Kazakh Autonomous Prefecture Animal Husbandry Station, Urumqi 835000, China; 13364712998@163.com (H.C.); m18095936088@163.com (R.Y.)

**Keywords:** Yili horse, specialized racing training, integrated analysis, multi omics

## Abstract

Yili horses undergo coordinated physiological adaptations across systems in response to customized training. This study aimed to clarify the molecular mechanisms of these adaptations by integrating analyses of cardiac function and multi-omics (lipidomics, transcriptomics, miRNomics). We collected whole blood samples from ten Yili horses before and after 12 weeks of specialized racing training to perform these analyses. Results showed training induced adaptive cardiac remodeling, with substantial increases in LVIDd and LVIDs. At the molecular level, this was accompanied by extensive blood lipid reprogramming (383 differential lipids), enriched in energy pathways like fatty acid metabolism. Transcriptomic analysis identified 851 differential genes, also enriched in energy-related pathways (e.g., oxidative phosphorylation). We constructed a miRNA–mRNA network (189 pairs), finding miRNAs such as miR-150 and miR-199b regulate key energy-supply mRNAs. Integrated analyses revealed precise modulation of pathways: (1) eca-miR-150 is associated with *AZIN1* and creatine, with potential links to arginine/proline metabolism; (2) miR-8903 is associated with *LRAT* and nicotinamide, with potential associations with vitamin absorption. These pathways are critical for energy metabolism, redox balance, and signal transduction. Overall, this study reveals how training optimizes energy supply and metabolic homeostasis in Yili horses, offering new insights into training adaptation physiology.

## Simple Summary

Long-term specialized racing training induces coordinated physiological adaptations across multiple systems in horses, crucial for enhancing athletic performance. However, the molecular mechanisms underlying these adaptations in Yili horses—an elite Xinjiang breed renowned for racing and endurance—remain largely unknown. This study aimed to explore the molecular mechanisms of such training-induced adaptations in Yili horses. We subjected ten Yili horses to 12 weeks of specialized racing training, analyzing changes in cardiac structure/function, transcriptome, lipidome, and miRNome. Post-training, cardiac parameters like left ventricular end-diastolic diameter (LVIDd) improved significantly. We identified 383 differential lipids, 851 differential genes, and an miRNA–mRNA regulatory network with 189 interaction pairs. Overall, this study elucidates training adaptation mechanisms in Yili horses, providing a foundation for evidence-based training strategies.

## 1. Introduction

Yili horses are a premium breed endemic to Xinjiang, China, and represent the first domestically developed racehorse in the country with independent intellectual property rights [1]. Recent studies indicate that equine explosive power, endurance, and recovery capacity are not solely determined by phenotypic selection; rather, systematic training activates multi-omics and multi-level regulatory networks, which in turn influence energy metabolism and muscle function to modulate these athletic traits [2,3]. However, as a representative high-performance horse breed combining endurance with explosive power, significant knowledge gaps remain regarding the molecular adaptive mechanisms underlying the athletic performance of Yili horses, as well as the regulatory differences in these mechanisms compared to other horse breeds (e.g., Thoroughbreds, Arabian horses). Meanwhile, gene and miRNA expression analyses alone can only suggest potential effects, whereas lipidomics, as the terminal layer of “omics”, directly reflects physiological endpoints. Over the past decade, most research has focused on endurance events [4]: during endurance exercise, metabolite levels (including lactic acid, amino acids, and lipids) undergo substantial changes, but studies on sprint exercises remain relatively limited [5,6]. Exercise training has been shown to induce changes in skeletal and cardiac muscle tissues, as well as in metabolism-related gene expression, thereby optimizing energy metabolism, muscle fiber composition, and cardiovascular function [7,8]. Nevertheless, how specialized racing training influences equine athletic performance through multi-level interactions involving miRNAs, mRNAs, and lipids has yet to be systematically elucidated.

In recent years, integrated multi-omics approaches have provided novel insights into the molecular mechanisms underlying exercise adaptation in horses. MicroRNAs (miRNAs), as critical post-transcriptional regulators, play a central role in exercise-induced systemic adaptation. Equine studies have shown that endurance or sprint training dynamically modulates the expression of miRNAs such as eca-miR-206, 133a/b, and 486-3p in the blood, which indirectly regulate muscle fiber type transformation and energy metabolism pathways by targeting key genes including *MSTN* and *SRF* [9,10]. Concurrently, messenger RNA (mRNA) expression profiling analyses in blood have revealed that exercise induces significant remodeling of gene networks associated with energy metabolism (e.g., glycolysis, fatty acid oxidation), inflammatory regulation, and tissue repair in horses. For instance, after sprint training in Thoroughbreds, the expression of blood transcripts of muscle function-related genes such as *MYH4* and *PGM1* is upregulated, whereas endurance training in Yili horses is characterized by changes in blood mRNA levels of metabolic regulatory genes including *CD36* and *PPARγ* [11,12]. Metabolomic studies further demonstrate that post-exercise metabolite levels in equine blood undergo substantial and specific changes: endurance training increases the content of energy metabolism intermediates such as branched-chain amino acids (BCAAs) and 3-hydroxybutyrate, while sprint training leads to dynamic fluctuations in lactate, creatine kinase, and sphingolipid metabolites. These blood metabolic signatures can directly reflect the skeletal muscle adaptive response to exercise [13,14].

Ten elite Yili horses were used as subjects in this study. By comparing the synergistic effects of whole-blood miRNA and mRNA expression profiles alongside metabolite levels between the pre-training group (Baseline Control, BC) and the specialized racing training-adapted group (Training-Adapted, TA), and integrating bioinformatics predictions with functional enrichment analyses, we systematically investigated the response mechanisms of metabolic networks underlying exercise. This multi-omics approach enabled reinforced integrative modeling across molecular layers. We hypothesize that elucidating the interactions among these three molecular components will allow us to: (i) characterize the complex interplay between the lipidome, transcriptome, and miRNome; and (ii) identify specific biomarkers associated with exercise-induced stress responses and improvements in performance.

## 2. Materials and Methods

### 2.1. Experimental Animals and Grouping

Ten healthy, superior Yili horses (five males and five females, 18 months old) were selected for this study. All horses had similar body measurements and weights and were housed under identical conditions (Supplementary Text S1). Prior to the experiment, the health of each horse was confirmed through physical examinations, lameness assessments, and echocardiography. Horses were maintained under uniform management, fed a standardized diet, and had free access to fresh water and salt licks. Each horse underwent a specific training program at a consistent intensity for three months. The pre-training period served as the Baseline Control Group (BC, *n* = 10), whereas the 3-month post-training period constituted the Training-Adapted Group (TA, *n* = 10). Notably, the lipidomics data for the TA group have been reported previously [15].

### 2.2. Blood Sample Collection and Preparation

Ten milliliters of jugular venous blood were collected from each horse before and after the training program and placed into EDTA-anticoagulated tubes. To control for circadian variation, all blood samples within the same training cycle were collected at the same time each day (8:00–9:00). High-intensity training was suspended 24 h prior to sampling to avoid acute exercise effects. Five milliliters of blood were mixed with TRIzol reagent at a 1:3 ratio for RNA extraction. The remaining 5 mL was centrifuged at 3000× *g* for 10 min to obtain plasma, which was immediately aliquoted into cryovials and stored at −80 °C until further analysis.

### 2.3. Echocardiographic Analysis

Following twice cleaning with 75% alcohol and coupling gel application on the right parasternal area of each horse, echocardiography was performed using a Mindray M6 veterinary ultrasound system (2.5 MHz phased-array probe; Mindray, Shenzhen, China) via the third to fifth intercostal spaces. The echocardiographic analysis protocol was performed as previously described in our published study [12]. Scans (2D and M-mode) were repeated three times non-consecutively with parameters: 30 cm depth, 5 mm focus, 110° sector angle, and pulse repetition frequency adjusted by blood flow velocity. Data were independently collected by a technician. Detailed procedures are in Supplementary Text S2.

### 2.4. Lipids Assessment and Analysis

#### 2.4.1. Data Collection and Processing

Blood samples (5 mL) were obtained from the jugular vein of each horse at rest. The samples were centrifuged at 3000× *g* for 10 min to separate plasma, which was then dispensed into cryovials and stored at −80 °C until subsequent analysis.

#### 2.4.2. Targeted Metabolomics Analysis

Metabolomics analysis was performed on an ultra-high-performance liquid chromatography-tandem mass spectrometry (UPLC-MS/MS) system. Following thawing, samples were vortexed briefly for 10 s to achieve thorough mixing. A 50 μL aliquot of each sample was pipetted into a labeled centrifuge tube, and 300 μL of 20% acetonitrile-methanol extraction solution containing internal standards was subsequently added. The samples were vortexed for 3 min, centrifuged at 15,000× *g* for 10 min at 4 °C, and 200 μL of the supernatant was transferred to a different labeled tube. After incubation at −20 °C for 30 min, the samples were centrifuged again at 15,000× *g* for 3 min at 4 °C, and 180 μL of the supernatant was transferred to a vial insert for UPLC-MS/MS analysis. Additional details are available in Supplementary Text S3.

#### 2.4.3. Metabolomics Data Analysis

Screening of differential metabolites was conducted via a combination of univariate and multivariate approaches, encompassing hypothesis testing, fold change (FC) analysis, PCA and orthogonal partial least squares discriminant analysis (OPLS-DA). The screening criteria were defined as variable importance in projection (VIP) > 1, *p*-value < 0.05. Using these criteria, metabolites with significant associations to training status were identified. Metabolite annotation was performed with the Kyoto Encyclopedia of Genes and Genomes (KEGG) pathway database. Additional detailed procedures are available in Supplementary Text S4.

### 2.5. Transcriptome Sequencing

#### 2.5.1. RNA Extraction and Quality Assessment

Total RNA (including mRNA) was extracted with TRIzol reagent per standard protocol. Concentration was measured by Qubit fluorometer, and quality by Qsep-400 analyzer (BiOptic, New Taipei, China), with samples of RIN > 7 selected. After purification, RNA quantity and purity were detected by NanoDrop ND-1000 (NanoDrop, Wilmington, NC, USA), and integrity was verified by Bioanalyzer 2100 (Agilent, Santa Clara, CA, USA). For miRNA extraction: 200 µL total RNA was processed via mirVana™ Kit (Thermo Fisher Scientific, Waltham, DE, USA) enrichment column, small RNAs eluted with 30 µL buffer, and fractions with peaks at 22~200 nt selected.

#### 2.5.2. mRNA Library Preparation and Sequencing

Total RNA was denatured with 3′ DNA adapters at 70 °C for 2 min, immediately chilled to room temperature, added with T4 RNA ligase 2 (NEB, M0530L), and ligated at 16 °C for ≥8 h. Residual adapters were removed by 5′-deadenylase + Rec J (RTP) at 37 °C for 30 min, followed by ligation of pre-adenylated 5′ adapters with T4 RNA ligase 1 (NEB, M0204L) at 37 °C for 60 min. Reverse transcription was performed with SuperScript II (Thermo, 18064014) at 50 °C for 60 min, 80 °C for 10 min to inactivate the enzyme. cDNA was amplified with Phusion Polymerase (NEB, M0530L): 98 °C pre-denaturation 30 s; 98 °C 10 s, 60 °C 30 s, 72 °C 15 s (10–16 cycles); 72 °C final extension 5 min. Products were purified and size-fractionated by 8% denaturing PAGE, then sequenced on Illumina HiSeq 2500 (Thermo Fisher Scientific, Waltham, DE, USA, single-end 50 bp, standard protocol).

#### 2.5.3. Quality Control and Data Comparison of mRNA Transcripts

Raw FASTQ [16] data were filtered and quality-controlled with fastp v0.23.2 (Q20 ≥ 97%, Q30 ≥ 93%, GC 50 ± 2%, adapter contamination < 0.05%, error rate ≤ 0.01%). Clean reads were aligned to the horse reference genome (GCF_002863925.1_EquCab3.0_genomic.fna) using HISAT2 2.2.1 [17] (coverage ≥ 90%), with alignment results visualized via IGV 2.18.4 [17]. Transcripts were assembled by StringTie 2.2.3 [18], and gene quantification was performed using FeatureCounts (Subread v2.0.1) to generate an FPKM-normalized expression matrix. Batch effects were corrected with ComBat-seq (R package sva 3.50.0). Differential expression analysis was conducted using DESeq2 v1.36.0 [19,20] with negative binomial GLMM. Genes with |log_2_FC| ≥ 1, *p* < 0.05, and FDR < 0.01 (Benjamini–Hochberg correction) were considered DEGs. PCA and Ward.D2 hierarchical clustering showed clear sample separation by time point without outliers.

#### 2.5.4. Functional Annotation and Pathway Analysis of Differentially Expressed mRNAs

Differentially expressed genes were subjected to Gene Ontology (GO) enrichment analysis using clusterProfiler 4.6.0 [21] and GO.db 3.16.0 in R 3.5.1. Significance thresholds were set at log_2_ fold change ≥ 1.5 and FDR < 0.05, with a minimum of five genes per pathway. KEGG pathway enrichment analysis was performed on the same set of DEGs using the same significance thresholds. KEGG database information can be found at the Kyoto Encyclopedia of Genes and Genomes (https://www.genome.jp/kegg accessed on 7 July 2025). GO and KEGG pathways with *p*-values < 0.05 were considered statistically significant and were visualized as charts and heatmaps.

### 2.6. miRNA Sequencing

#### 2.6.1. miRNA Isolation, cDNA Library Construction, and Sequencing Identification

The <200 nt small RNA fraction from Section 2.4 was reanalyzed with the Agilent Small RNA Kit, confirming >80% of the major peak corresponded to 22 nt miRNAs. Library construction used 50 ng RNA input with the TruSeq Small RNA Library Prep Kit (Illumina, San Diego, CA, USA, RS-200-0012). Illumina-compatible adapters (RA3, RA5) were ligated to the 3′ and 5′ ends of miRNA fragments, respectively. First-strand cDNA was synthesized by reverse transcription, followed by 12 PCR cycles. Library fragments (140–160 bp) were size-selected and purified via 6% TBE polyacrylamide gel electrophoresis. Reads were aligned to mRNA, RFam, and Repbase databases to remove non-miRNA sequences. Remaining sequences were processed for miRNA identification using ACGT101-miR v4.2, with candidates annotated and validated against miRBase and reference genome annotations. Differential expression analysis was performed at *p* < 0.01 to identify DE-miRNAs.

#### 2.6.2. Differential Expression Analysis and Target Gene Prediction of miRNAs

Differential expressions between the 3-month and 6-month training periods were evaluated using edgeR v3.38 [22] with a quasi-likelihood F-test. The design matrix included Time as a fixed effect, Sex as a covariate, and a random effect of (1|Horse_ID). miRNAs with |log_2_FC| ≥ 1 and FDR < 0.01 were considered significantly differentially expressed [23,24]. Target genes of DE-miRNAs were predicted by integrating three complementary approaches: (i) TargetScan [25], requiring a context++ score percentile ≥ 50 [26]; (ii) miRanda [27], with a maximal binding free energy ≤ −10 kcal/mol [28]; and (iii) RNAhybrid, confirming secondary structural stability with *p* < 0.05 [29]. Only target genes identified by all three methods were retained for downstream analyses.

#### 2.6.3. Correlation Analysis of Differentially Expressed miRNA-mRNA Targets

High-confidence target genes of DE-miRNAs were intersected with differentially expressed mRNAs (|log_2_FC| ≥ 1, FDR < 0.01) from transcriptome analysis to identify putative differentially expressed targets. Normalized expression values of miRNAs and their corresponding target mRNAs were subjected to Pearson correlation analysis. Significant negative correlations were defined as r ≤ 0 and *p* < 0.05, yielding a set of significantly negatively correlated miRNA–mRNA pairs. An interaction network was constructed using iGraph v1.3.0 in R v4.0.4 and visualized with a spherical layout. Genes within network modules were subjected to GO and KEGG enrichment analyses using clusterProfiler v4.4.4, with significance set at FDR < 0.05 [30].

#### 2.6.4. Weighted Gene Co-Expression Network Analysis

Weighted Gene Co-Expression Network Analysis (WGCNA) was employed to investigate the relationships between genes and core metabolites. Gene co-expression networks were constructed using the WGCNA v1.69 [31] package in R. Pearson correlation matrices (with a threshold of R ≥ 0.75 and *p* ≤ 0.05) were utilized to establish associations between signature genes and core metabolites, with parameters set to default values. The co-expression networks were visualized using Cytoscape v3.10.1 [32]. Hub genes in the networks were identified based on degree centrality. Subsequently, correlation analysis was performed between the top 15 core metabolites and the top 15 core genes.

### 2.7. RT-qPCR Validation of mRNA and miRNA

The transcriptomic data employed in this study were derived from a 2023 research project. This project centered on two primary objectives: first, investigating the effects of long-term exercise on cardiac structure and function in horses; second, performing profiling analyses of blood transcriptomes and plasma lipidomes. Comprehensive details concerning sample preparation methods, experimental treatment procedures, and the study’s background have been previously documented in relevant research publications.

All samples incorporated in the current study underwent processing following standard protocols, subsequent to sequencing. Furthermore, preliminary validation of these samples was conducted using quantitative polymerase chain reaction (qPCR). It is important to note that no residual sample material remained available for additional validation experiments.

The raw sequencing data produced in this study have been deposited in the Sequence Read Archive (SRA) database maintained by the National Center for Biotechnology Information (NCBI), under the accession number SUB15457665. For complete protocols encompassing sample preparation, sequencing operations, and qPCR verification processes, please refer to the dissertation [33].

### 2.8. Statistical Analysis

All graphs were generated using GraphPad Prism 8.0 (GraphPad Software Inc, San Diego, CA, USA). Statistical analysis of the data was performed using SPSS 26.0 (IBM, Armonk, NY, USA). Data were expressed as mean ± standard deviation. Differences between groups were analyzed using evaluated using one-way ANOVA or Student’s *t*-test. Statistical significance was set at ns *p* > 0.05; * *p* < 0.05; ** *p* < 0.01. Homogeneity of variance within groups was tested, with *p* > 0.05 indicating no significant difference.

## 3. Results

### 3.1. Structural and Functional Indicators of the Left Ventricle in Yili Horses

**Figure 1:**
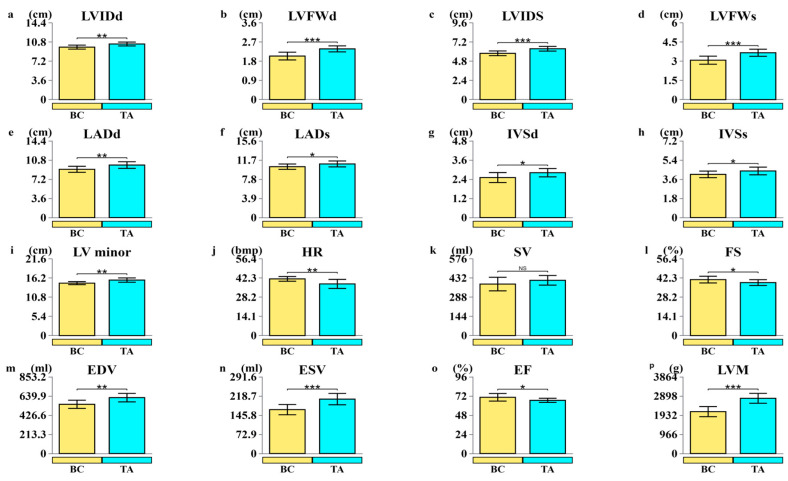
Variations in the anatomical and functional characteristics of the left ventricle before and after specific training. BC: Baseline Control Group; TA: Training-Adapted Group. (**a**) LVIDd: end-diastolic left ventricular diameter; (**b**) LVFWd: end-diastolic left ventricular free wall thickness; (**c**) LVIDs: end-systolic left ventricular diameter; (**d**) LVFWs: end-systolic left ventricular free wall thickness; (**e**) LADd: end-diastolic left atrial diameter; (**f**) LADs: end-systolic left atrial diameter; (**g**) IVSd: end-diastolic interventricular septal thickness; (**h**) IVSs: end-systolic interventricular septal thickness; (**i**) LV minor: internal diameter of the left ventricle; (**j**) HR: heart rate; (**k**) SV for stroke volume; (**l**) FS for fractional shortening; (**m**) EDV: end-diastolic left ventricular volume; (**n**) ESV: end-systolic left ventricular volume; (**o**) EF stands for ejection fraction; (**p**) LVM: left ventricular myocardial mass. *: *p* < 0.05, **: *p* < 0.01, ***: *p* < 0.001.

Prior to training initiation, we looked at the left ventricle of Yili horses and discovered that the BC and TA groups differed significantly in LVIDd, LVFWd, LVIDs, LADd, LADs, IVSd, IVSs, LV minor, HR, SV, FS%, EDV, ESV, EF%, and LVM. Although SV markers showed changes, they did not reach statistical significance (Figure 1).

### 3.2. Metabolomic Differences Before and After Specialized Racing Training

**Figure 2:**
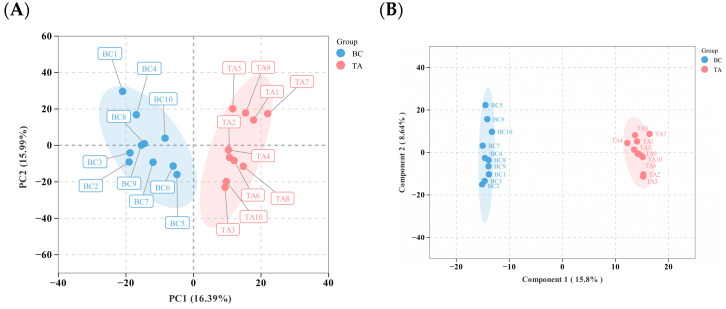

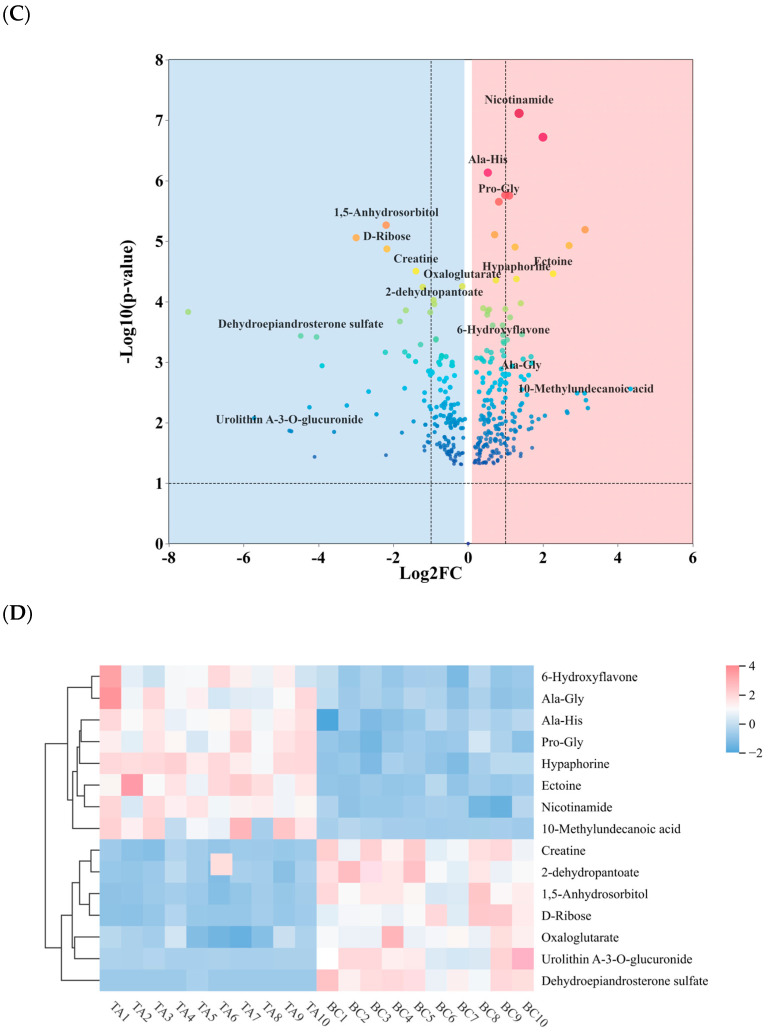

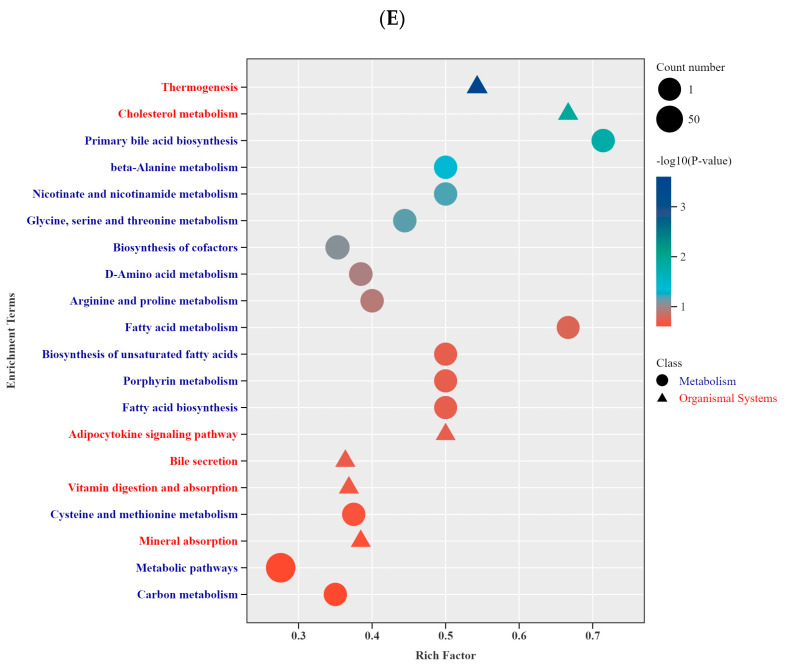
(**A**) Principal Component Analysis (PCA) plot. PC1 denotes the first principle components, PC2 denotes the second principle components, and percentages indicate the proportion of variance in the dataset explained by PC1 and PC2. Each point in the graphic represents a sample; samples from the same group have the same color. “Group” denotes grouping; the same applies below. (**B**) A score plot developed using Orthogonal Partial Least Squares Discriminant Analysis (OPLS-DA). The predictive principal component is represented by the horizontal axis, whose direction indicates differences between groups; The orthogonal principal component is represented by the vertical axis, whose direction indicates intra-group differences; percentages show how well each component explains the dataset. Each point in the graphic represents a sample, with samples from the same group colored identically. (**C**) Volcano plot of differential lipids, where labeled lipids are core lipids with VIP values greater than two and significance to the research topic. (**D**) Heatmap of core differential lipids. (**E**) KEGG Pathway Enrichment Analysis of Differential lipids.

Principal component analysis (PCA) of all differential lipids showed tight clustering of quality control samples, confirming the stability of the LC-MS platform. Biological replicates within each group exhibited consistent clustering and high reproducibility, while a clear separation was observed between the BC and TA groups, indicating significant alterations in metabolite accumulation patterns following specialized racing training (Figure 2A). All samples from both groups fell within their respective 95% confidence ellipses, with well-defined boundaries between groups. Orthogonal partial least squares discriminant analysis (OPLS-DA) further confirmed the robustness and reliability of the model, showing no evidence of overfitting and accurately representing sample variation (Figure 2B).

A total of 383 differentially expressed lipids (DEMs) were identified, including 220 upregulated and 162 downregulated lipids (Figure 2C). Further screening highlighted 15 key lipids with VIP > 2 and strong relevance to the study, which are displayed in a heatmap (Figure 2D): Creatine, 6-Hydroxyflavone, Pro-Gly, Dehydroepiandrosterone sulfate, Nicotinamide, Hypaphorine, Ectoine, Ala-His, 10-Methylundecanoic acid, Ala-Gly, Urolithin A-3-O-glucuronide, 2-dehydropantoate, Oxaloglutarate, D-Ribose, and 1,5-Anhydrosorbitol. KEGG pathway enrichment analysis of the DEMs revealed significant overrepresentation in pathways including primary bile acid biosynthesis, beta-alanine metabolism, cholesterol metabolism, and thermogenesis (Figure 2E).

### 3.3. Functional Classification and Annotation of Differentially Expressed mRNAs

#### Functional Classification and Annotation of Differentially Expressed Genes

**Figure 3:**
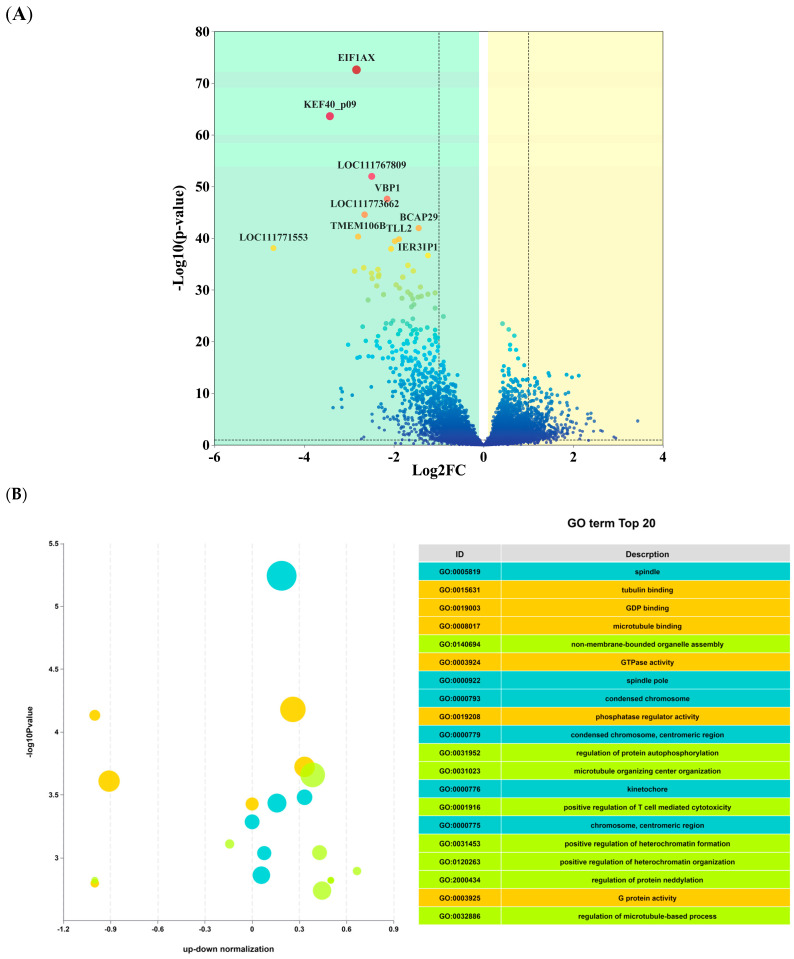

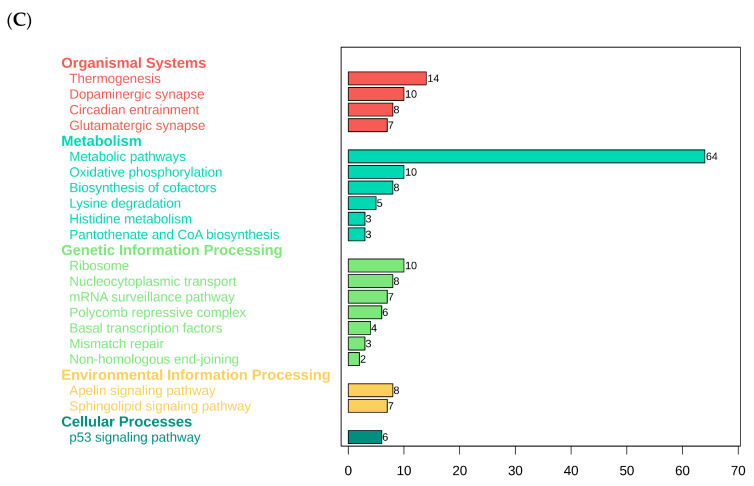
(**A**) Volcano plot of differentially expressed genes between the two groups. (**B**) GO enrichment circle plot of differentially expressed genes. (**C**) KEGG bubble plot of differentially expressed genes.

Using |log2Fold Change| ≥ 1 and FDR < 0.05 as screening criterion, 851 differentially expressed genes (DEGs) were found across the two groups, including 509 downregulated genes and 342 upregulated genes (Figure 3A).

To explore the potential biological functions of the differentially expressed genes (DEGs), Gene Ontology (GO) enrichment analysis was conducted. A total of 43 secondary GO terms were significantly enriched, spanning the three main categories: biological process (BP), molecular function (MF), and cellular component (CC). The top 20 enriched GO terms are presented in Figure 3B. To further illustrate pathway enrichment, a KEGG bubble plot was generated for the top 20 pathways. The results indicated that these DEGs were predominantly involved in pathways associated with energy metabolism, exercise endurance, neuromuscular regulation, and exercise adaptation signaling, including oxidative phosphorylation, metabolic pathways, thermogenesis, dopaminergic synapse, glutamatergic synapse, apelin signaling pathway, and p53 signaling pathway (Figure 3C).

### 3.4. Differential miRNA Sequencing and Target Gene Functional Analysis

**Figure 4:**
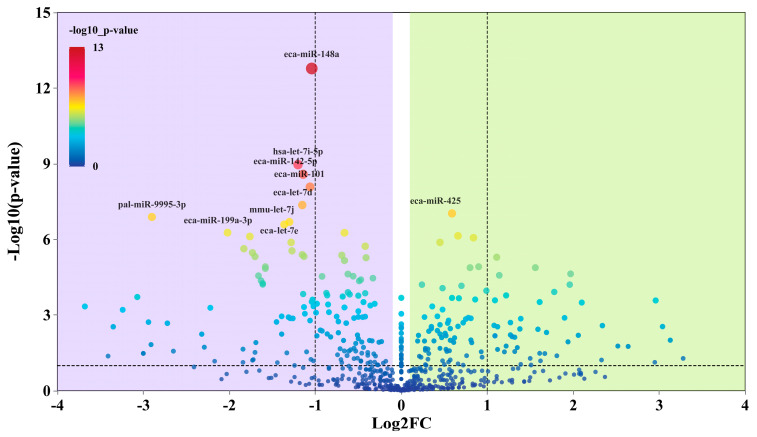
Volcano plot of significantly differentially expressed miRNAs between groups A and B.

A total of 259 differentially expressed miRNAs (DE-miRNAs) were identified between the BC and TA groups. Differential expression was defined as |log_2_FC| > 1 and *p* < 0.01. Among these, 81 miRNAs were significantly altered, including 29 upregulated miRNAs such as eca-miR-150 and eca-miR-129a-3p, and 52 downregulated miRNAs including eca-miR-21, eca-miR-132, eca-miR-8903, eca-miR-148a, and eca-miR-199a-3p (Figure 4).

### 3.5. Integrated miRNA–mRNA Regulatory Network Reveals Upstream Regulatory Factors

**Figure 5:**
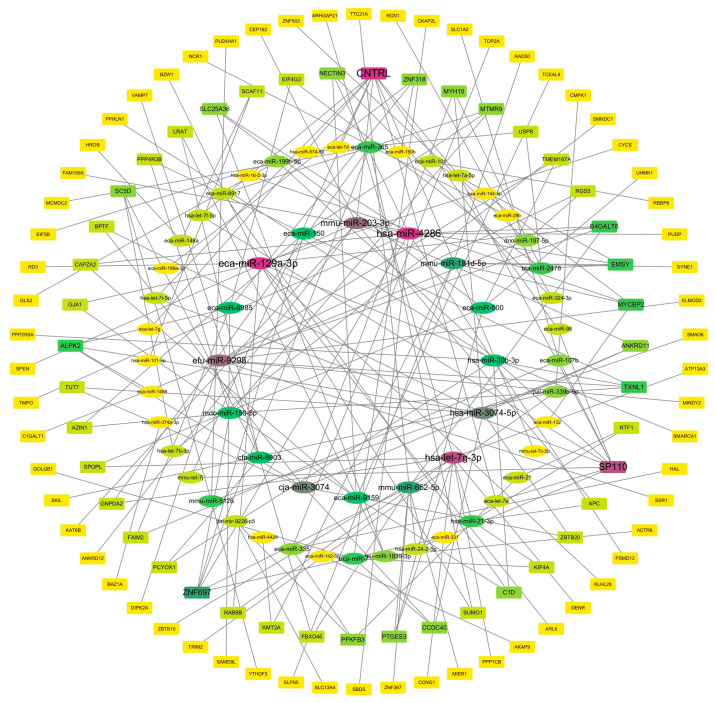
Interaction network between miRNAs and their target genes. (The deeper the color, the higher the connectivity.)

Target genes of DE-miRNAs were predicted using TargetScan 7.0 and miRanda 3.3a, and then intersected with differentially expressed mRNAs. Pearson correlation analysis was performed to identify negatively correlated pairs. This analysis yielded a regulatory network consisting of 189 significantly negatively correlated miRNA–mRNA pairs (Figure 5).

### 3.6. WGCNA Identification of Hub Genes Associated with Core Lipids

**Figure 6:**
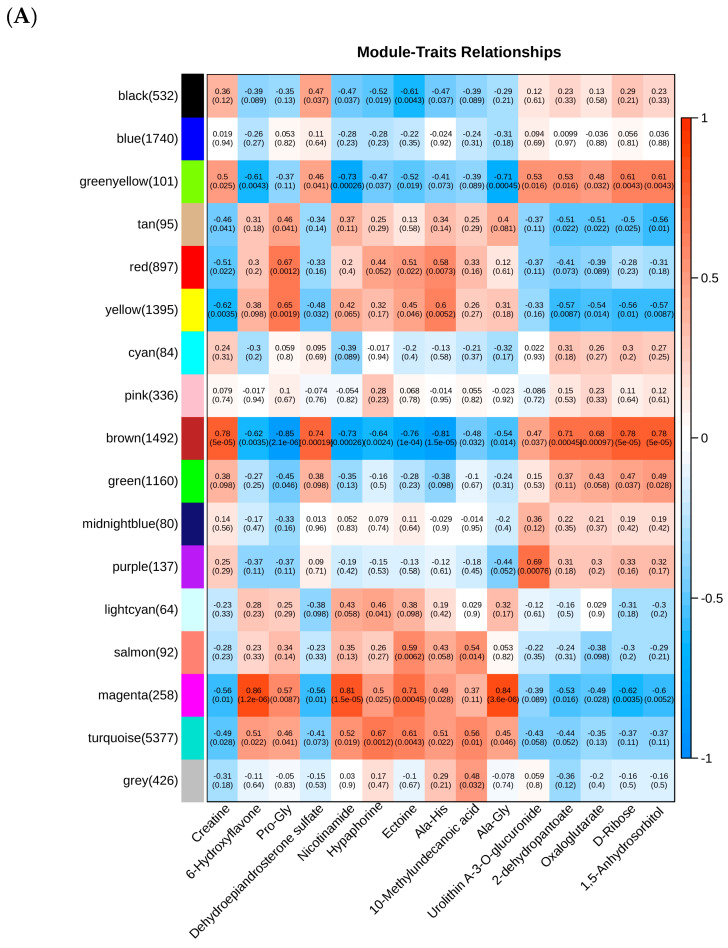

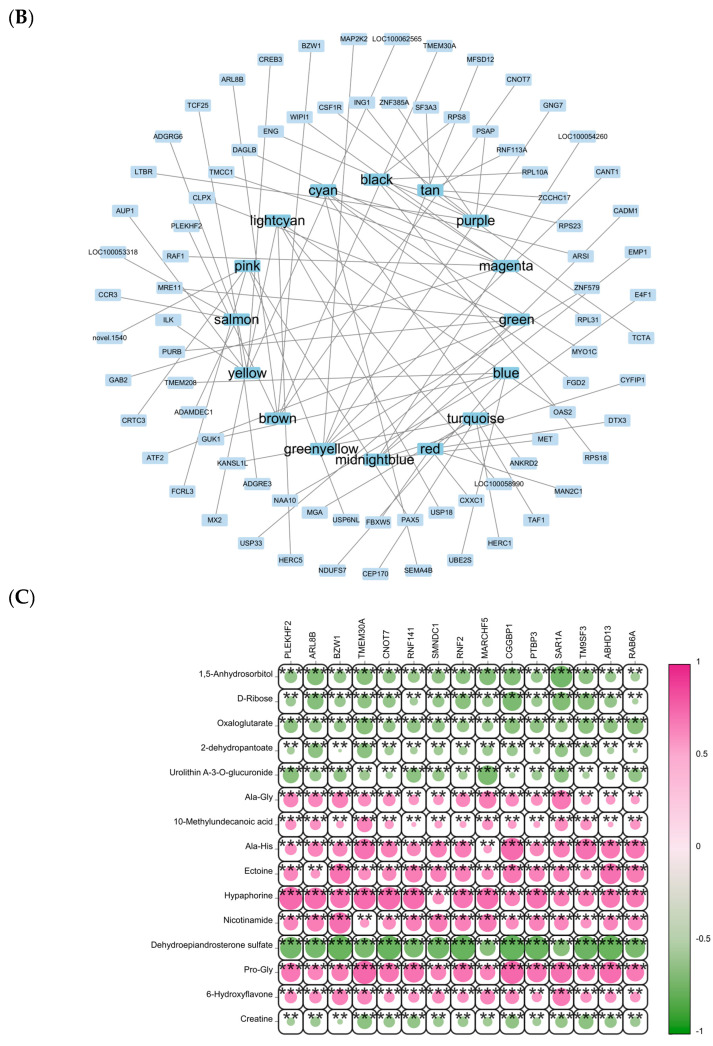
WGCNA analysis findings. (**A**) Core gene expression levels and core metabolite concentrations in relation to each other. One module and one metabolite are represented by each row and column, respectively. (**B**) Co-expression network of learned modules and genes. (**C**) Correlation analysis between key metabolites and key genes. (Green indicates a negative correlation, and red indicates a positive correlation). ** *p* < 0.01, *** *p* < 0.001.

The following key lipids were used as trait files: Creatine, 6-Hydroxyflavone, Pro-Gly, Dehydroepiandrosterone sulfate, Nicotinamide, Hypaphorine, Ectoine, Ala-His, 10-Methylundecanoic acid, Ala-Gly, Urolithin A-3-O-glucuronide, 2-dehydropantoate, Oxaloglutarate, D-Ribose, and 1,5-Anhydrosorbitol. WGCNA was performed using a soft threshold of β = 16 (Supplementary Text S5A,B). The resulting unscaled network satisfied goodness-of-fit criteria, and average connectivity was examined across different power levels. Hierarchical clustering identified 17 gene modules, with strong co-expression observed among genes within each module (Figure 6A). The top five hub genes in each module were visualized using Cytoscape (Figure 6B). Among these, the brown module exhibited strong correlations with the core lipids and was selected for further integrative analysis between candidate genes and lipids (Figure 6C).

### 3.7. Multi-Omics Integrated Analysis to Construct a Synergistic Regulation Model for Training Adaptation

**Figure 7:**
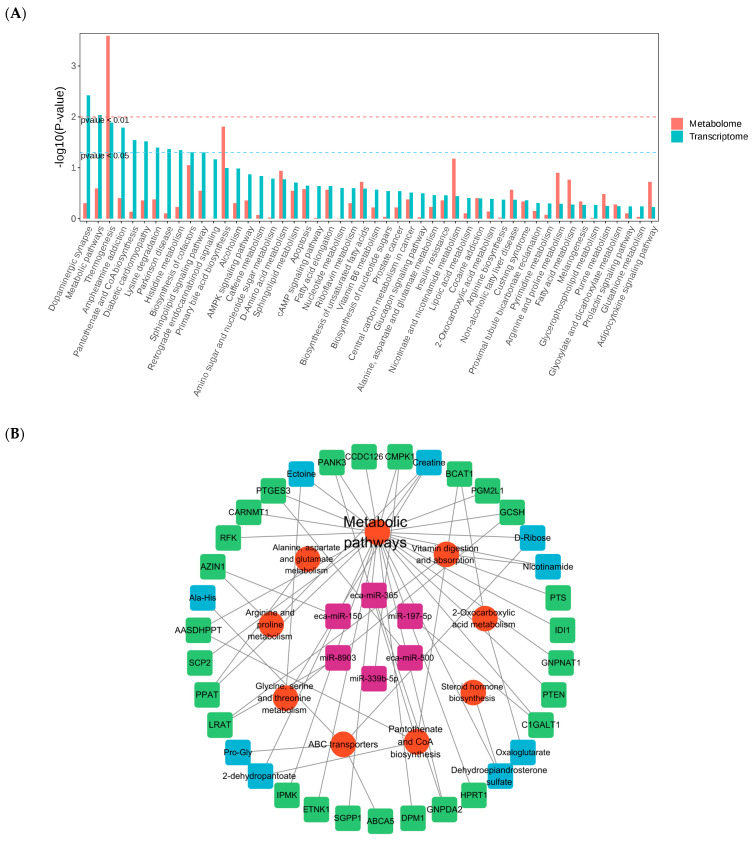

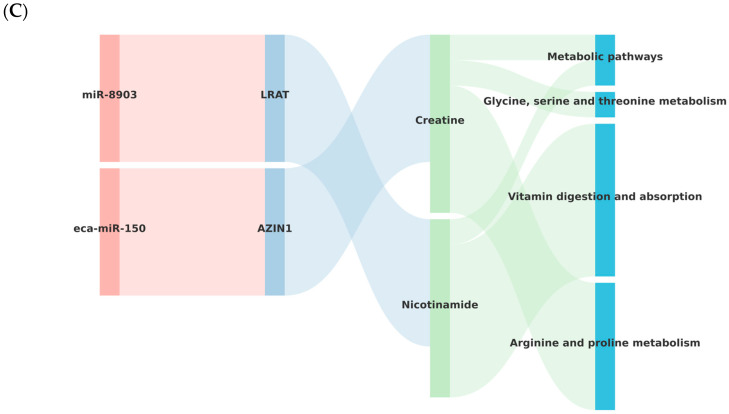
(**A**) Bar plot of integrated transcriptome-metabolome KEGG co-enrichment pathway analysis (The *x*-axis represents the names of KEGG pathways, the *y*-axis represents the statistical significance *p*-value of enrichment in the corresponding pathway, and the red and green colors indicate the metabolome and transcriptome, respectively.) (**B**) Core regulatory network illustrating interactions among miRNAs, key DEGs, DEMs, and pathways. Colors represent different components: purple–miRNAs, red–pathways, green–mRNAs, blue–metabolites. (**C**) Mechanism diagram of core miRNAs, mRNAs, metabolites, and pathways (the thicker the connecting line, the greater the relevance/importance).

Pathway associations were assessed using KEGG enrichment analysis for both differentially expressed genes (DEGs) and differentially expressed lipids (DEMs) (Figure 7A), with significance evaluated based on *p*-values. The integrated analysis revealed that, compared with the control group (BC), the training-adapted group (TA) exhibited significant enrichment in key pathways, including metabolic pathways, biosynthesis of cofactors, and nucleotide metabolism.

The brown module from WGCNA and core lipids were utilized to investigate pathway enrichment (Figure 7B). This analysis identified two core potential regulatory axes characterized by associations: (1) eca-miR-150 is associated with *AZIN1* and Creatine, with potential links to arginine and proline metabolism; (2) miR-8903 is associated with *LRAT* and Nicotinamide, with potential associations with vitamin digestion and absorption (Figure 7C).

## 4. Discussion

Peripheral venous blood provides a detectable molecular expression profile that not only transports oxygen and nutrients but also reflects training-induced changes in gene expression profiles, regulatory networks, functional modules [34,35], and metabolic remodeling (e.g., dynamic alterations in lipids such as lactate, branched-chain amino acids, acylcarnitines, and ketone bodies) via circulating RNAs [36,37]. Building on observed changes in cardiac structural and functional markers, this study further integrated mRNA, miRNA, and metabolite analyses. The results indicate that molecules including eca-miR-129a-3p, BZW1, Creatine, and Nicotinamide, as well as pathways related to thermogenesis, primary bile acid biosynthesis, and cholesterol metabolism, play key roles in mediating the physiological response to training stimuli.

### 4.1. Structural and Functional Analysis of the Yili Horse Heart

Numerous studies have demonstrated a strong correlation between heart size and athletic performance in horses, particularly in racehorses. The larger cardiac capacity of thoroughbred racehorses is often cited as a key factor contributing to their superior competitive performance. This anatomical advantage allows for increased cardiac output and enhanced oxygen delivery under high physiological loads, supporting exceptional endurance and speed [38]. Following specialized racing training, cardiac anatomy exhibited clear modifications compared with pre-training measurements. The most pronounced changes included enlargement of the left ventricular end-diastolic diameter (LVIDd) and thickening of the left ventricular end-systolic wall (LVIDs), indicative of improved myocardial contractility and higher stroke volume. These adaptations enable the heart to meet physiological demands with less effort at rest, providing direct evidence of optimized cardiac function [39]. Additionally, heart rate decreased markedly after training. Long-term athletes, especially endurance-trained individuals, commonly exhibit sinus bradycardia, as a larger stroke volume allows the heart to maintain adequate circulation without increasing beat frequency. Collectively, these findings suggest that the Yili horse heart undergoes substantial physiological remodeling in response to specialized racing training.

### 4.2. Metabolomic Differences in BC and TA Under Specialized Racing Training Conditions

Regular physical exercise has been shown to exert numerous beneficial effects on metabolic health [40]. Further analysis of lipidomics data (Figure 2D) identified core lipids involved in key physiological processes, including energy metabolism and muscle function, fatty acid oxidation and metabolism, oxidative stress and anti-inflammatory responses, and amino acid and peptide metabolism. Creatine, a central component of energy metabolism, increases muscular phosphocreatine stores during high-intensity exercise such as running, enabling rapid ATP regeneration to meet energy demands [41]. D-Ribose primarily contributes to energy metabolism and recovery by serving as a precursor for ATP, thereby facilitating ATP resynthesis to maintain energy supply during exercise. Observed post-training reductions in D-Allulose suggest that prolonged exercise enhances mitochondrial performance and fatty acid oxidation, reducing reliance on carbohydrates (including D-Ribose) and increasing the use of fatty acids for energy. This adaptation decreases the requirement for D-Ribose consumption or synthesis. Nicotinamide, a form of vitamin B3, acts as a precursor for NAD+ and is essential for energy metabolism, cellular signaling, and antioxidant defense. Studies indicate that nicotinamide influences cellular energy metabolism by regulating the mTOR and AMPK signaling pathways, which are critical for maintaining cellular energy homeostasis and mitochondrial function [42].

KEGG pathway analysis revealed post-training lipid enrichment, predominantly in pathways related to fatty acid metabolism, biosynthesis of unsaturated fatty acids, fatty acid production, and carbon metabolism. Fatty acid oxidation enhances athletic performance, while carbon metabolism supports rapid energy provision, suggesting that long-term training optimizes horses’ energy supply. Additional enriched pathways, such as vitamin digestion and absorption, mineral absorption, and cysteine and methionine metabolism, indicate that training also improves the metabolic efficiency of vitamins, minerals, and amino acids.

### 4.3. Combined miRNA-mRNA Analysis of BC and TA Under Specific Training Conditions

Based on the miRNA–mRNA co-analysis, overexpression of miR-150 (log_2_FC = 1.08) directly suppresses *AZIN1*. Previous studies indicate that miR-150 mitigates adverse remodeling and fibrosis following myocardial infarction by targeting pro-fibrotic genes such as Hoxa4 and apoptosis-related genes like *Gdap1l1*, thereby improving cardiac function [43]. In the heart, *AZIN1* primarily regulates fibrosis and regenerative repair. It alleviates cardiac fibrosis by inhibiting the TGF-β/Smad3 signaling pathway and promotes cardiomyocyte proliferation through circRNA–miRNA regulatory axes. Furthermore, suppression of its splice variant *AZIN2-sv* has been reported to enhance angiogenesis and cardiac function after myocardial ischemia [44].

The findings also demonstrate that downregulation of miR-199b-5p (log_2_FC = 2.32) increases *GLS2* expression and enriches pathways related to glutamine metabolism, protein binding, and mitochondrial function. Prior research has shown that reduced miR-199b-5p expression decreases inflammatory cell infiltration in rat myocardium, attenuates endoplasmic reticulum stress in myocardial infarction models, and improves myocardial fibrosis and cardiac function [45]. Its primary roles involve the regulation of cardiac hypertrophy, fibrosis, and inflammation [46]. *GLS2*, a mitochondrial p53 target gene, participates in antioxidant defense and metabolic reprogramming, enhancing the TCA cycle via α-ketoglutarate (aKG) and promoting efficient ATP synthesis [47]. Additionally, miR-8903 (log_2_FC = 1.22) was found to target *LRAT*. Under hypoxic conditions, the expression of *GPR146* may be co-regulated by multiple miRNAs, including miR-8903, miR-11972, and miR-466x. It has been suggested that miR-8903 may modulate *GPR146* upregulation, thereby contributing to adaptive responses under hypoxia [48]. In the heart, *LRAT* serves as a key indicator of vitamin A storage, and its downregulation is associated with primary hypertension and adverse cardiac remodeling. Reduced *LRAT* expression may lead to vitamin A deficiency, which can affect the renin–angiotensin system, promoting myocardial hypertrophy, ventricular fibrosis, and impaired cardiac function [49].

miR-7 plays a crucial role in neuroendocrine regulation and tumor-suppressive processes. However, emerging evidence suggests that elevated miR-7 expression may serve as a potential therapeutic target for DM1-associated muscle dysfunction by promoting myoblast fusion and myotube hypertrophy, thereby alleviating muscle atrophy [50]. *TOP2A*, whose enriched pathways indicate involvement in DNA replication and repair (GO:0006259), is associated with cell proliferation and muscle regeneration when miR-335 is downregulated (log_2_FC = 3.68). Its connection to the p53 signaling pathway (KEGG:04115) suggests that exercise may stimulate myogenic satellite cell proliferation or tissue repair via the miR-335–*TOP2A* axis. Tomé et al. [51] initially reported that miR-335 induces p53-dependent cell cycle arrest by targeting *RB1*, thereby inhibiting DNA repair and reducing proliferative activity. In a 5-month resistance training study on elderly participants, decreased miR-335 expression was correlated with a higher proportion of Ki-67^+^ satellite cells, indicating that training reverses miR-335-mediated suppression to facilitate muscle regeneration [52]. Functionally, *TOP2A* modulates double-strand break formation, DNA binding, and topological DNA structure, thereby regulating transcription, replication, and associated cellular processes. It also influences oxidative phosphorylation efficiency and controls cell proliferation via the AKT/mTOR pathway [53]. These findings suggest that negative regulation of the miR-335/*TOP2A* axis plays a critical role in coordinating cell cycle control, DNA repair, and energy metabolism, representing an important mechanism underlying exercise adaptation and muscle regeneration.

### 4.4. Combined Transcriptome-Lipidome Analysis of BC and TA Under Specific Training Conditions

Integrated transcriptomic–metabolomic analysis revealed that, compared with pre-training levels, post-training groups exhibited significant enrichment in pathways related to thermogenesis, primary bile acid biosynthesis, and cholesterol metabolism. These changes reflect multisystem metabolic adaptations and energy homeostasis remodeling induced by exercise. The enrichment of thermogenesis pathways directly supports the notion that long-term exercise increases energy expenditure. Exercise not only stimulates energy consumption through skeletal muscle activity but also promotes thermogenesis and energy regulation via multiple physiological processes. For instance, studies have shown that exercise enhances whole-body energy expenditure by promoting the browning of white adipose tissue, a process whereby white adipocytes are converted into thermogenic “beige” adipocytes, which is markedly amplified during exercise [54]. One study investigated the effects of combined exercise training and dietary weight loss on hepatic bile acid production in obese women. After approximately 14 weeks, fasting serum total bile acid levels decreased by roughly 30% in sedentary, insulin-resistant participants, while fasting serum 7-alpha-hydroxy-cholest-3-en-4-one (C4), a marker of *CYP7A1* enzyme activity, increased by 55% [55]. These findings suggest that exercise and dietary interventions can modulate bile acid metabolism, and improved metabolic health is associated with enhanced bile acid production during fasting. The relationship between cholesterol metabolism and exercise is complex and multifaceted. Moderate aerobic exercise has been shown to positively influence cholesterol metabolism by activating the PPAR signaling pathway, thereby improving lipid transport and energy expenditure [56]. Additionally, exercise can modulate cholesterol metabolism by regulating markers of cholesterol absorption and biosynthesis. Although an 8-week aerobic program did not significantly reduce serum total cholesterol, it exhibited a trend toward lower indicators of cholesterol absorption [57].

In this study, multi-omics analyses identified key roles for miR-150 and miR-8903, which regulate *AZIN1* and *LRAT*, respectively, and are associated with creatine and nicotinamide enrichment in the arginine and proline metabolism and vitamin digestion and absorption pathways. Under exercise or stress conditions, proline dehydrogenase (PRODH)-mediated proline catabolism contributes intermediates to the tricarboxylic acid (TCA) cycle, enhancing ATP production and restoring glutathione redox balance, thereby counteracting cardiac remodeling [58]. Arginine, as a precursor for nitric oxide (NO) synthesis, can improve coronary perfusion and endothelial function, enhance cardiomyocyte survival, and, in combination with exercise training, delay the progression of heart failure [59]. Furthermore, arginine and proline metabolism interacts with phosphocreatine metabolism to modulate the turnover of high-energy phosphate compounds in the myocardium, further regulating cardiac contractile function and mitigating the risk of pathological remodeling [60]. The digestion and absorption of vitamins ensure sufficient nutrient availability, which is critical for maintaining oxidative stress balance, energy metabolism, and overall cardiovascular health, particularly under physiological stress induced by exercise [61].

Our findings suggest that this small-sample-size approach is feasible, as the biological effects induced over medium- to long-term training are sufficiently robust to produce stable signaling alterations detectable in blood. We further demonstrate that quantitative analysis of a limited set of key lipids can sensitively reflect overall changes in energy metabolism, oxidative stress, fatty acid oxidation, anti-inflammatory responses, as well as amino acid and peptide metabolism. Although complex regulatory layers and temporal dynamics may prevent metabolite levels from directly or immediately mirroring transcriptional changes, we were able to validate the physiological relevance of the observed metabolic alterations by examining the correlations between transcriptional levels and the concentrations of substrates and products within the corresponding metabolic pathways.

This study has limitations: due to ethical constraints on animal use and sample collection challenges, plasma-based lipidomic and transcriptomic analyses cannot fully reflect the direct gene expression and metabolic changes in myocardial tissue during training-induced cardiac remodeling. Currently, studies on whether changes in plasma mRNA and lipid metabolites can accurately mirror those in cardiomyocytes remain scarce. Although existing research has indirectly confirmed via animal models and clinical samples that certain plasma gene products, metabolites such as lysophosphatidylcholine, are associated with cardiac fibroblast expression or cardiac function and can serve as biomarkers [62,63,64], clear evidence for their direct correspondence is lacking, further highlighting the limitations of using plasma as a surrogate tissue in this study.

## 5. Conclusions

This study reveals the distinct regulatory mechanisms underlying metabolic adaptation and performance enhancement in Yili horses, demonstrating a clear regulatory cascade. Training first induces adaptive remodeling of cardiac structure and function, evidenced by significant increases in LVIDd and LVIDs, accompanied by a reduction in heart rate, suggesting improved ventricular pumping efficiency. At the molecular level, metabolomic analysis identified differentially expressed lipids enriched in energy metabolism pathways, while transcriptomic analysis showed that differentially expressed genes were predominantly involved in energy-related processes, including oxidative phosphorylation, AMPK signaling, and thermogenesis. By constructing a miRNA–mRNA regulatory network, we identified 189 strongly negatively correlated regulatory pairs. Integrated multi-omics analysis further highlighted two core regulatory associations: (1) eca-miR-150, which exhibits a regulatory association with *AZIN1*, interacts with creatine, and may thereby be involved in modulating arginine and proline metabolism; and (2) miR-8903, which shows a regulatory association with *LRAT*, is associated with nicotinamide, and may be involved in vitamin digestion and absorption. These findings provide molecular-level insights into the associations underlying how phase-specific training optimizes energy supply and nutrient metabolism homeostasis in horses, offering a novel perspective on the physiological basis of training adaptation.

## Data Availability

The original contributions presented in the study are included in the article/Supplementary Material, further inquiries can be directed to the corresponding authors.

## Supplementary Materials

The following supporting information can be downloaded at: https://www.mdpi.com/article/10.3390/biology14111609/s1, Supplement Text S1 Horse body measurements and feeding conditions; Supplement text S2: Echocardiographic Analysis; Supplement Text S3: Lipid LC-MSMS Analysis Experimental Method; Supplement Text S4: Metabolomics analysis Method; Supplement Text S5: WGCNA.

## Abbreviations

The following abbreviations are used in this manuscript:

HR	Heart rate
EF	Ejection fraction
FS	Fractional shortening
LVIDd	End-diastolic left ventricular diameter
LVIDs	End-systolic left ventricular diameter
IVSd	End-diastolic interventricular septal thickness
IVSs	End-systolic interventricular septal thickness
LVM	Left ventricular myocardial mass
LVFWd	End-diastolic left ventricular free wall thickness
LVFWs	End-systolic left ventricular free wall thickness
LVLD	Left ventricle long axis diameter
LADd	End-diastolic left atrial diameter
LADs	End-systolic left atrial diameter
EDV	End-diastolic left ventricular volume
ESV	End-systolic left ventricular volume
SV	Stroke volume
