# Correction: Wang et al. Multi-Omics Analysis Reveals Biaxial Regulatory Mechanisms of Cardiac Adaptation by Specialized Racing Training in Yili Horses. *Biology* 2025, *14*, 1609

**DOI:** 10.3390/biology15030209

**Published:** 2026-01-23

**Authors:** Tongliang Wang, Mengying Li, Wanlu Ren, Jun Meng, Xinkui Yao, Hongzhong Chu, Runchen Yao, Manjun Zhai, Yaqi Zeng

**Affiliations:** 1 College of Animal Science, Xinjiang Agricultural University, Urumqi 830052, China; wtl13639911402@163.com (T.W.); 17613305908@163.com (M.L.); renwanlu@xjau.edu.cn (W.R.); junm86@xjau.edu.cn (J.M.); yaoxinkui@xjau.edu.cn (X.Y.); zhaimanjun@yeah.net (M.Z.); 2 Xinjiang Key Laboratory of Horse Breeding and Exercise Physiology, Urumqi 830052, China; 3 Horse Industry Research Institute, Xinjiang Agricultural University, Urumqi 830052, China; 4 Xinjiang Yili Kazakh Autonomous Prefecture Animal Husbandry Station, Urumqi 835000, China; 13364712998@163.com (H.C.); m18095936088@163.com (R.Y.)

**Error in Figure**

In the original publication [1], there was a mistake in Figure 2B as published. When we submitted the higher-resolution images of the figures, we mistakenly inserted the image of Figure 2A in place of Figure 2B. As a result, the image of Figure 2A appears twice in the published version. The corrected Figure 2B appears below. The authors state that the scientific conclusions are unaffected. 

This correction was approved by the Academic Editor. The original publication has also been updated. This correction was approved by the Academic Editor. The original publication has also been updated.

## Figures and Tables

**Figure 2:**
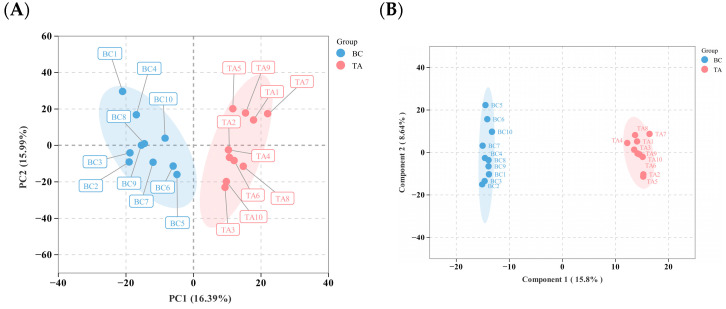

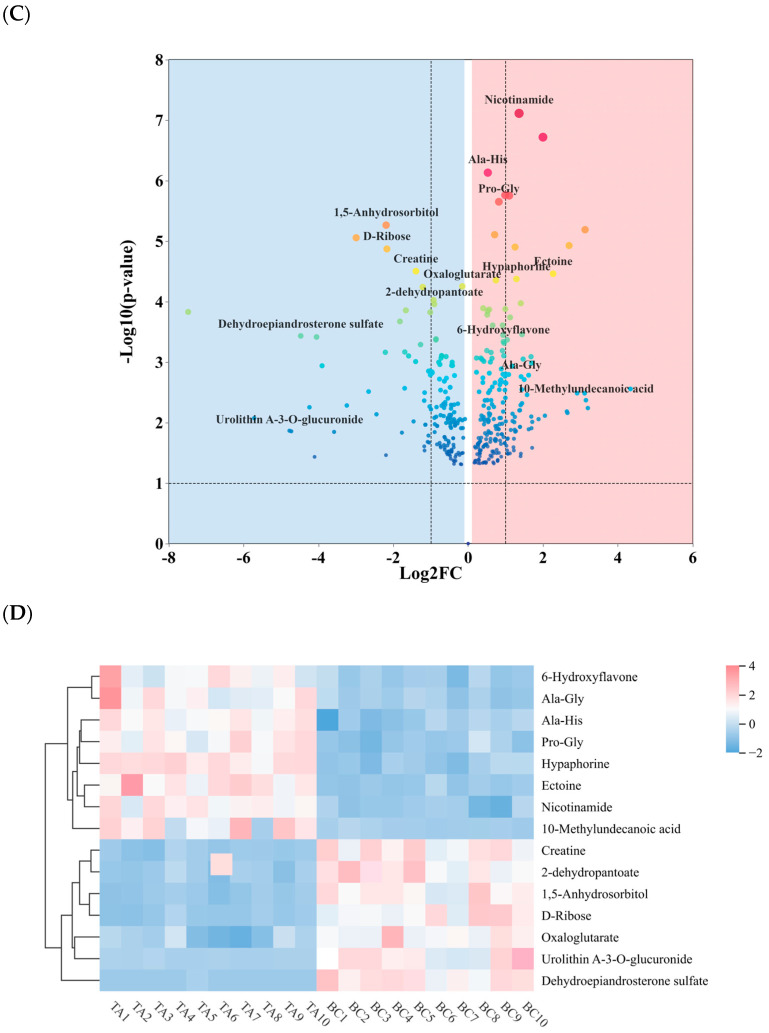

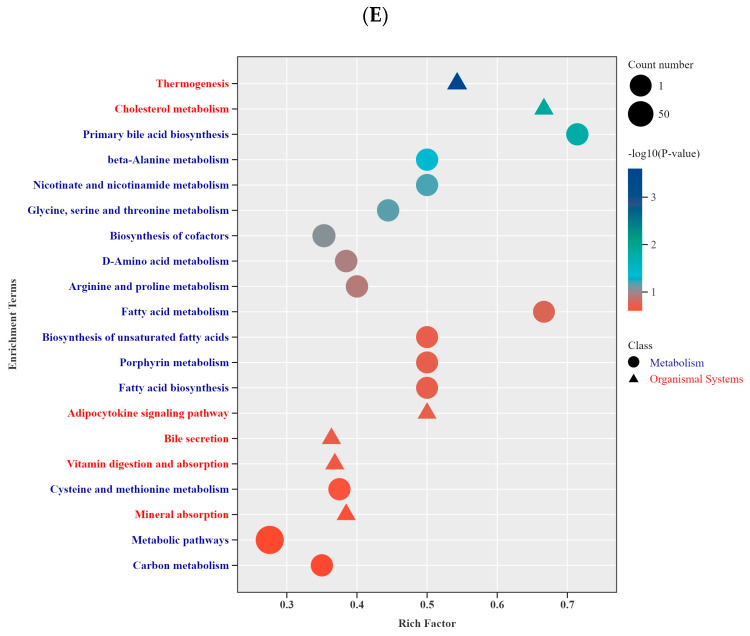
(**A**) Principal Component Analysis (PCA) plot. PC1 denotes the first principle components, PC2 denotes the second principle components, and percentages indicate the proportion of variance in the dataset explained by PC1 and PC2. Each point in the graphic represents a sample; samples from the same group have the same color. “Group” denotes grouping; the same applies below. (**B**) A score plot developed using Orthogonal Partial Least Squares Discriminant Analysis (OPLS-DA). The predictive principal component is represented by the horizontal axis, whose direction indicates differences between groups; The orthogonal principal component is represented by the vertical axis, whose direction indicates intra-group differences; percentages show how well each component explains the dataset. Each point in the graphic represents a sample, with samples from the same group colored identically. (**C**) Volcano plot of differential lipids, where labeled lipids are core lipids with VIP values greater than two and significance to the research topic. (**D**) Heatmap of core differential lipids. (**E**) KEGG Pathway Enrichment Analysis of Differential lipids.
