# Supplementary material for: Multi-Omics Analysis Reveals Biaxial Regulatory Mechanisms of Cardiac Adaptation by Specialized Racing Training in Yili Horses
Source: Biology (Basel). 2025 Nov 17;14(11):1609. doi: 10.3390/biology14111609 (PMC12649962; doi:10.3390/biology14111609)
Supplement: Supplementary file 1 [file biology-14-01609-s001.zip › Supplement Text 1 Horse body measurements and feeding conditions.pdf]

Supplement text 1

Each horse was fed 8 kg/d of dry forage and 4 kg/d of concentrate supplement.

Table S1. Composition and nutritional levels of the basal diet (on dry matter basis).

Item	Content (%)
**Ingredients**	
Corn	17.28
Wheat bran	5.26
Soybean meal	9.26
Monocalcium phosphate	1.15
Salt	0.63
Premix	0.31
Methionine	0.19
Dry forage	65.92
Total	100
**Nutritional Levels**	
Dry matter	95.31
Crude protein	12.69
Crude fat	1.77
Neutral detergent fiber	42.23
Acid detergent fiber	37.98
Crude ash	8.16
Calcium	0.86
Phosphorus	0.41
**Digestible energy (MJ/kg)**	9.03

Note: 1. The premix provided the following per kg of the concentrate supplement: VA 14 mg, VB1 21.29 mg, VB2 336.5 mg, VB6 1.27 mg, VD 2.3 mg, VE 857 mg, biotin 6 mg, pantothenicacid 4.56 mg, nicotinamide 12.17 mg, Cu (as copper sulfate) 43.24 mg, Fe (as ferrous sulfate) 111.94 mg, Mn (as manganese sulfate) 183.27 mg, Zn (as zinc sulfate) 176.04 mg, I (as potassium iodide) 29.69 mg, Se (as sodium selenite) 42.29 mg, Co (as cobalt chloride) 4.06 mg.

2. Nutrient levels were measured values.

Table S2. Body measurements of horses before training.

Group	withers height	body length	chest circumference	circumference of cannon bone
BC1	147	144	166	18
BC2	145	142	158	17
BC3	146	142	160	17.5
BC4	143	140	159	17
BC5	143	138.5	150	17

BC6	146	140	154	17
BC7	143	142	159	17
BC8	146	142	161	17.5
BC9	147	144	160.5	16
BC10	146	146	162	17.5
