# Supplementary material for: Multi-Omics Analysis Reveals Biaxial Regulatory Mechanisms of Cardiac Adaptation by Specialized Racing Training in Yili Horses
Source: Biology (Basel). 2025 Nov 17;14(11):1609. doi: 10.3390/biology14111609 (PMC12649962; doi:10.3390/biology14111609)
Supplement: Supplementary file 1 [file biology-14-01609-s001.zip › Supplement Text 2 Echocardiographic Analysis.pdf]

Supplement text 2

The right parasternal examination area of each horse was cleaned twice with 75% medical alcohol, and both the probe and the examination area were coated with coupling gel. Echocardiographic measurements were performed using a Mindray M6 veterinary ultrasound system equipped with a 2.5 MHz phased-array probe. Standardized cardiac scanning was conducted through the right parasternal acoustic window, spanning the third to fifth intercostal spaces. Imaging parameters were set as follows: maximum penetration depth of 30 cm, dynamic focus positioned at 5 mm depth, sector scan angle of 110°, and pulse repetition frequency adjusted according to blood flow velocity. Each observation plane, including both 2D and M-mode views, was scanned three times in a non-consecutive manner. All echocardiographic data were independently collected by a certified ultrasound technician.

Specific Operating Protocols

Plane Acquisition: The left ventricular outflow tract was visualized using the right parasternal long-axis plane (B-mode), while dynamic images of the short-axis plane (B/M composite mode) were acquired simultaneously. Data from both end-diastolic and end-systolic phases were saved in DICOM format.

Measurement Protocol: Parameters were measured over three non-consecutive cardiac cycles (corresponding to a heart rate of 32–50 beats per minute). For each parameter, the arithmetic mean of two valid measurements was calculated.

Parameter Extraction: Sixteen cardiac assessment indices were quantitatively obtained from the sequence of right parasternal short-axis images.
