# Supplementary material for: Multi-Omics Analysis Reveals Biaxial Regulatory Mechanisms of Cardiac Adaptation by Specialized Racing Training in Yili Horses
Source: Biology (Basel). 2025 Nov 17;14(11):1609. doi: 10.3390/biology14111609 (PMC12649962; doi:10.3390/biology14111609)
Supplement: Supplementary file 1 [file biology-14-01609-s001.zip › Supplement Text 3 Lipid LC-MSMS Analysis Experimental Method.pdf]

Supplement text 3

First Category Liquid Samples

Samples stored at –80°C were thawed on ice and vortexed for 10 seconds. To a 2 mL microcentrifuge tube, 50 µL of the sample was added along with 300 µL of extraction solvent (acetonitrile:methanol = 1:4, v/v) containing internal standards. After vortexing for three minutes, the mixture was centrifuged at 12,000 rpm for 10 minutes at 4°C. Subsequently, 200 µL of the supernatant was collected, placed at –20°C for 30 minutes, and centrifuged again at 12,000 rpm for 3 minutes at 4°C. Finally, 180 µL of the supernatant was transferred for LC-MS analysis.

Chromatographic Conditions

All samples were analyzed using three liquid chromatography–mass spectrometry (LC-MS) methods. For the first analysis, samples were evaluated in positive ion mode using a T3 column (Waters ACQUITY UPLC HSS T3 C18, 1.8 µm, 2.1 mm × 100 mm) with a mobile phase of 0.1% formic acid in water (A) and 0.1% formic acid in acetonitrile (B). The gradient elution was set as follows: 5% to 20% B over 2 minutes, then to 60% B over 3 minutes, followed by 99% B for 1 minute, held for 1.5 minutes, and returned to 5% B over 0.1 minutes. The total phase B duration was approximately 2.4 minutes. Column temperature was maintained at 40°C, flow rate at 0.4 mL/min, and injection volume at 2 µL or 5 µL. For the second analysis, samples were analyzed in negative ion mode using the same elution gradient as the positive mode. The third analysis employed negative ionization with a HILIC column (Waters ACQUITY UPLC BEH HILIC, 1.7 µm, 1 mm × 150 mm). The mobile phase consisted of solvent A: 60% acetonitrile, 30% water, and 10% methanol containing 20 mM ammonium carbamate, pH 10.6; and solvent B: 40% acetonitrile and 60% water with 20 mM ammonium carbamate. The gradient was as follows: 5% to 30% B over 3.5 minutes, 50% to 95% B over 2 minutes, held at 95% B for 1 minute, and then returned rapidly to the initial conditions.

QTOF-MS/MS

Data acquisition was performed in information-dependent acquisition (IDA) mode using Analyst TF 1.7.1 software (Thermo Fisher Scientific, Concord, Ontario). The source parameters were set as follows: ion source gas 1 (GAS1), 50 psi; ion source gas 2 (GAS2), 50 psi; curtain gas (CUR), 25 psi; temperature (TEM), 550°C; dissociation potential (DP), 60 V or –60 V for positive or negative mode, respectively; ion spray voltage floating (ISVF), 5,000 V or –4,000 V for positive or negative mode, respectively.

For the TOF-MS scan, the parameters were: mass range 50–1,000 Da; accumulation time 200 ms; dynamic background subtraction enabled. For the product ion scan, the parameters were: mass range 25–1,000 Da; accumulation time 40 ms; collision energy 30 V or –30 V in positive or negative mode, respectively; collision energy spread, 15; resolution, unit; mass-to-charge ratio, 1:1; intensity threshold, 100 s/min; isotope exclusion within 4 Da; mass tolerance, 50 ppm; maximum number of candidate ions per cycle, 18.

ESI-Q TRAP-MS/MS

A triple quadrupole-linear ion trap mass spectrometer (QTRAP) equipped with an ESI source was used to perform LIT and triple quadrupole (QQQ) scans. The system (QTRAP® LC-MS/MS) operated in both positive and negative ion modes and was controlled by Analyst 1.6.3 software (Sciex). The ESI source parameters were as follows: source temperature, 500°C; ion spray voltage (IS), 5,500 V for positive ions and –4,500 V for negative ions; source gas I (GSI) and gas II (GSII), 50 psi each; curtain gas (CUR), 25 psi; collision gas (CAD), set to high. Instrument calibration and

mass calibration were performed using 10 and 100 µmol/L ethyl polyacrylate solutions in QQQ and LIT modes, respectively. For each time interval, a specific set of multiple reaction monitoring (MRM) transitions was monitored based on the lipids eluting during that period.
