# Supplementary material for: Multi-Omics Analysis Reveals Biaxial Regulatory Mechanisms of Cardiac Adaptation by Specialized Racing Training in Yili Horses
Source: Biology (Basel). 2025 Nov 17;14(11):1609. doi: 10.3390/biology14111609 (PMC12649962; doi:10.3390/biology14111609)

WGCNA

(A) Soft threshold for gene co-expression, with the x-axis representing β values and the y-axis representing the scaling network model exponent. (B) Clustering dendrogram of gene co-expression modules, where the major branches form 16 modules labeled with various colors.

A

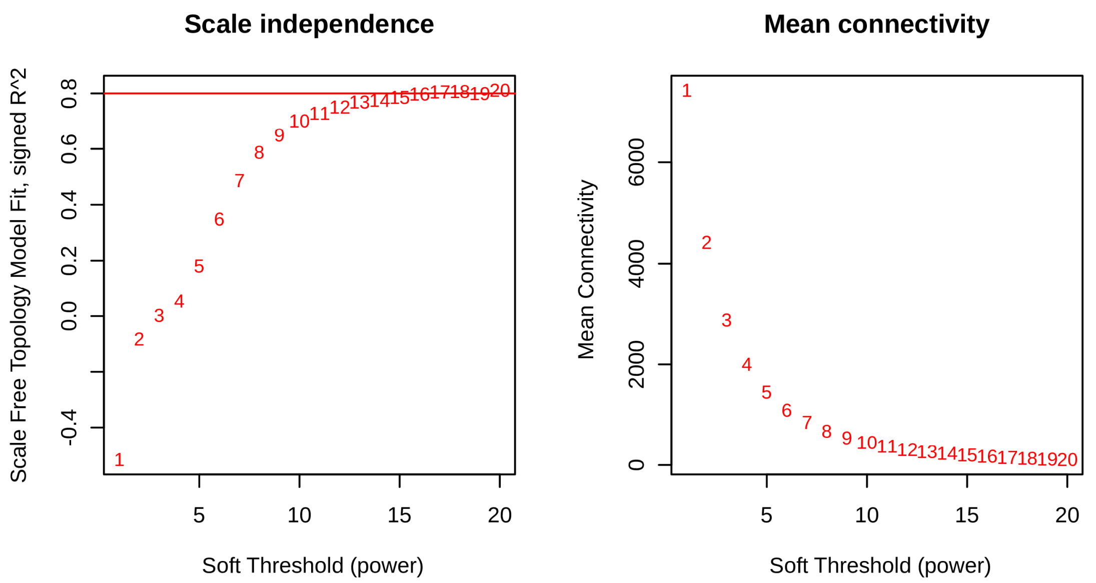

B

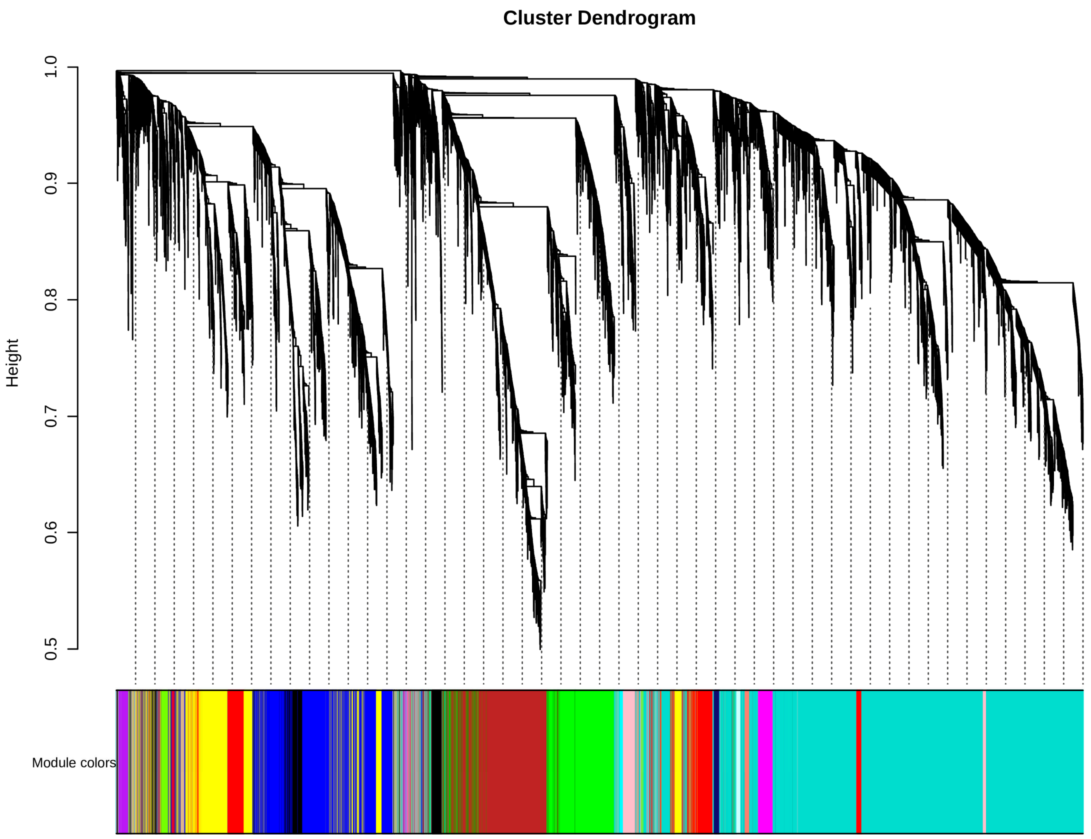

Supplement: Supplementary file 1 [file biology-14-01609-s001.zip › Supplement Text 5 WGCNA .pdf]
